# Impact of Defocus and High-Order Aberrations on Light Disturbance Measurements

**DOI:** 10.1155/2019/2874036

**Published:** 2019-01-02

**Authors:** A. Amorim-de-Sousa, R. Macedo-de-Araújo, P. Fernandes, A. Queirós, J. M. González-Méijome

**Affiliations:** Clinical Experimental Optometry Research Laboratory (CEORLab), Center of Physics, School of Sciences, University of Minho, Braga, Portugal

## Abstract

**Purpose:**

To evaluate the impact of different levels of positive and negative defocus on light disturbance (LD) measures and to understand how high-order aberrations (HOAs) and topographic quality parameters may influence the perception of photic phenomena.

**Methods:**

Thirty young healthy subjects (21 females and 9 males) attended this cross-sectional study. LD was evaluated with the light distortion analyzer (LDA) in natural accommodative and cycloplegic conditions with positive and negative induced defocus of 1.00D. HOAs were taken for a natural mesopic (without cycloplegia) and for fixed 5 mm (with cycloplegia) pupil size. The impact of corneal morphological parameters (SAI, SRI, and Q-value) in LD was also investigated.

**Results:**

Positive and negative induced defocus of 1.00D significantly increased the size of LD (*p* < 0.010, Wilcoxon signed rank test) but not its irregularity index. Spherical-like HOAs were associated with the size of LD, while coma-like and total-like HOAs were associated with LD irregularity. Our results showed that SRI was significantly correlated with the size of the disturbance area (*r*=0.519, *p*=0.003, Spearman correlation) and SAI with both size (*r*=0.502, *p*=0.005, Spearman correlation) and irregularity (*r*=0.371, *p*=0.044, Spearman correlation). However, no correlation between the Q-value and LD parameters was found.

**Conclusions:**

The uncorrected positive and negative refractive errors might increase the size of the LD, such as the spherical-like HOAs, SAI, and SRI, instead of asphericity. Coma-like and total-like HOAs and SAI may influence the perception of irregularities in the LD shape. These results might have an impact on postrefractive surgery visual performance that should be investigated.

## 1. Introduction

Visual disturbances affect people's performance in everyday activities, especially in low lighting conditions. Intraocular light scattering, intrinsic ocular aberration, and uncorrected refractive errors affect the vision quality by degrading the retinal image. The degradation is more relevant at night when the pupil is dilated, and objects are seen against a dark background, which leads to the perception of dysphotopsias or disturbances around bright light sources [1–4]. Ageing [5, 6], ocular pathologies [1, 7, 8], contact lens wear [9], and corneal treatments [10–12] can change ocular scattering and high-order aberrations (HOAs). Subjects with an affected visual quality usually manifest a decrease in contrast sensitivity and complain about poor night vision due to a higher perception of light disturbances (photic phenomena) [4, 13–17]. According to Jabbur et al. [18], the most subjective complaints of dissatisfied patients after a refractive surgery are blurred distance vision (59.0%), glare, and night vision disturbances (43.5%).

Uncorrected refractive errors are a leading cause of visual impairment in a significant proportion of the general population, if they are either undiagnosed or improperly corrected [19–21]. Some studies [22–24] showed that defocus affects the driving performance, especially at night when ocular aberrations have a major impact. Macedo-de-Araújo et al. [25] reported that light disturbance (LD) increased with the induction of more positive spherical-like aberrations, while negative spherical-like HOAs had no impact on the perceived LD. The increase in LD perception with positive induced defocus has already been reported to be significant over +1.00D of defocus [8, 26]. However, few studies evaluated the differences between the positive and negative defocus on LD [27, 28].

There is an important coupling effect between the defocus and HOAs that might be relevant for improving visual performance. However, there is no consensus about how HOA and refractive errors interact. Some studies [29, 30] reported that the combination of positive spherical-like HOA and the induction of negative defocus may produce a better visual performance. Other researchers showed no correlation between the HOA and spherical ametropias and no differences between the ametropic and emmetropic eyes on the spherical-like HOA magnitude [31, 32].

Different methodologies have been proposed and described to quantify the retinal image quality, but only a few were approved and validated [1, 13, 27, 33, 34]. The light distortion analyzer (LDA, CEORLab, University of Minho, Portugal) allows measuring the size, shape, and irregularity of LD without a video display. This device consists of a central LED surrounded by 240 smaller LEDs equally distributed over 24 meridians and provides different metrics to evaluate LD under more realistic conditions than other devices [33, 35]. LDA showed to be sensitive to small changes in HOA [25] and on evaluating LD in subjects who underwent refractive treatments [16, 36].

The importance and need of correcting low refractive errors should not be dismissed, not only for the improvement of visual acuity but also for the visual quality under dim light conditions. This study evaluated the impact of low positive and negative induced defocus on LD perception under low luminance conditions, as well as the effect of HOA, corneal surface asymmetry index (SAI) and surface regularity index (SRI), and asphericity (Q-value) on such photic phenomena.

## 2. Methods

### 2.1. Sample

In this cross-sectional experimental study, 30 healthy subjects (18 to 40 years of age) took part. Inclusion criteria were the spherical refractive error between +2.00 and −3.00D, astigmatism below 1.50D, and less than 1.00D of anisometropia. Subjects should present a best-corrected VA of 0.00 LogMAR units or better in each eye and the difference between eyes less than 0.1 LogMAR units. Transparent ocular media with no ocular pathology or previous ocular surgery were required, and they should not take any ocular or systemic drugs with ocular affectation.  Table 1 presents the characteristics of the sample. The protocol was approved by the Ethics Subcommittee for Life and Health Sciences and follows the guidelines of the Declaration of Helsinki. After explaining the objectives and procedures of the study, all subjects signed an informed consent.

### 2.2. Procedure

High (100%) and low (10%) contrast visual acuities were measured with Logarithmic Visual Acuity Chart ETDRS (Precision Vision, IL) at 4 meters after a full optometric examination to assess suitability to enter the study.

Aberrometry and LD measures were carried out under natural and cycloplegia conditions. Cycloplegia was obtained by topical instillation of two drops of Tropicil top 10 mg/ml (Edol, Portugal). The IRx3 Hartmann-Shack aberrometer (ImaginEyes, France) was used to obtain the total HOAs up to the sixth order, expressed as Zernike polynomials. Total (corneal and internal) HOA RMS (from Z_3_^−3^ to *Z*_6_^6^) and spherical-like (*Z*_4_^0^ and *Z*_6_^0^) and coma-like HOA RMS (Z_3_^−1^, *Z*_3_^1^, Z_5_^−1^, and *Z*_5_^1^) were considered. All HOAs were derived for the natural pupil size during the measurement of LD (noncycloplegic conditions) and for a 5 mm pupil diameter when in cycloplegia.

LD was monocularly (right and left eyes) and binocularly evaluated with the LDA under natural (baseline) and cycloplegic conditions. Cycloplegic measurements were randomly taken with the best distance vision correction and with positive and negative induced defocus (+1.00D and −1.00D, respectively). An in-out 30° routine exam was used: a peripheral LED was presented from the center to periphery in 12 semimeridians with an angular separation of 30°, surrounding the central LED. Subjects were asked to maintain their fixation in the central LED (source of glare) during the exam and performed some training before starting measurements. Under cycloplegia, all subjects used a 5 mm artificial diaphragm centered with the pupil at 12 mm from the corneal vertex, to standardize the pupil size. The room illumination while evaluating LD was 0.78 ± 0.03LUX.

In this study, we evaluated size (LDI and BFCRad) and irregularity (BFCIrreg and BFCIrregSD) parameters of LD. The light disturbance index (LDI) is the percentage of the total tested area that is not visible due to LD impairment (ratio of the area missed by the subject to the total area explored). The best-fit circle (BFC) is the one that best fits the outermost area of disturbance, with a radius equal to the average length of the disturbance along each semimeridian (BFCRad), centered at *X*, *Y* coordinates corresponding to the geometric centroid of the disturbance area. BFCIrreg (best-fit circle irregularity) is the sum of positive and negative deviations between the disturbance area and the BFC along the semimeridians tested, and BFCIrregSD is the sum of differences squared and divided by the numbers of semimeridians tested, which suggests the irregularity of the disturbance [33]. The area of analysis in the LDA device covers a circle of 16 cm, which at a distance of 2 meters represents a visual angle of 4.58°. As a reference, a 100% LDI value will correspond to 4.58 degrees of field, and 80 mm would be the maximum expected BFCRadius.

The hardware characterization has been described in an earlier paper [35]. The instrument has been tested for intrasession and intersession repeatability in a clinical context, using different disturbance intensities, examination strategies, different pupil sizes, and with different polar resolutions. Median values and interquartile ranges showed that the device had a good test-retest consistency when measured under pupil diameters of 3 and 6 mm, for low, medium, and maximum central glare source intensity [33]. The in-out random exam protocol with a 30° polar resolution showed comparable results to longer exam protocols including 15° polar resolution, and for that reason, this protocol is used in the present study and in other previous clinical evaluations involving patients implanted with intraocular lenses [36]. Further information on the device setup, hardware radiometric characterization, and validation can be found elsewhere in the referred publications [33, 35].

SAI, SRI, and asphericity (Q-value) of the anterior corneal surface were assessed with Medmont E300 (Medmont Pty, Ltd, Melbourne, Australia) before cycloplegic instillation. The natural pupil size was measured with the NeurOptics® VIP™-200 Pupillometer (Irvine, California, USA) in the same illumination conditions of LD measurements without cycloplegia. All measures were made in one visit only.

Statistical analysis was performed with SPSS Statistic software version 23.0 (SPSS Inc, Chicago, IL). The descriptive data are presented in mean ± standard deviation. The normality of all variables was evaluated using the Kolmogorov–Smirnov test. The paired samples *t*-test was used to compare variables with normal distribution and the Wilcoxon paired test for those who do not fulfil this assumption of normality. Spearman correlation was considered strong if >0.800, moderate if between 0.500 and 0.800, fair if between 0.300 and 0.500, and poor if <0.300 [37]. The level of significance was set at *α* = 0.050. Only one eye (randomly chosen) was considered for monocular evaluation since both eyes were strongly correlated. Further details on the methodology can be checked in reference [38].

## 3. Results

### 3.1. Light Disturbance Analysis

All monocular LD parameters were found to be higher than the binocular ones in all measuring conditions (baseline, with cycloplegia, +1.00D defocus, and −1.00D defocus). Size parameters (LDI and BFCRad) showed statistically significant differences (*p* ≤ 0.011, Wilcoxon paired samples test) between defocus conditions. Regarding irregularity parameters, only BFCIrreg in noncycloplegic conditions showed a statistically significant difference between the monocular and binocular values (*p*=0.004, Wilcoxon paired samples test). Cycloplegic values were always higher than baseline (*p* ≤ 0.019, Wilcoxon paired samples test), except for irregularity parameters in monocular conditions (*p* ≥ 0.141, Wilcoxon paired samples test).

**Figure 1:**
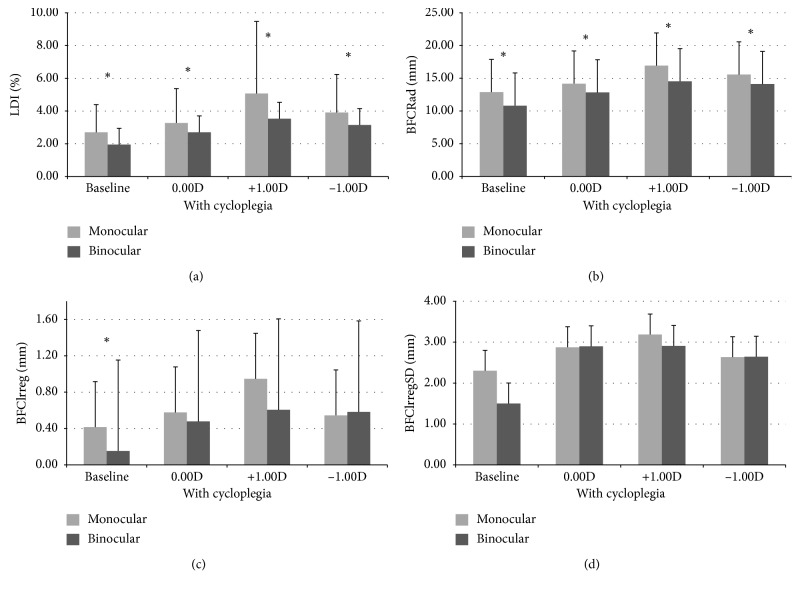
Monocular and binocular LDA size (graphs (a) and (b)) and irregularity (graphs (c) and (d)) values (mean ± standard deviation) represented in barplots for the four conditions evaluated: baseline (natural accommodative conditions and pupil size) and in cycloplegia with zero, myopic and hyperopic induced spherical defocus (0.00D, +1.00D and −1.00D, respectively) with 5 mm pupil (corresponding to 0.14° or 8.01 minarc). (∗) Statistically significant differences.

Concerning the impact of defocus on LD measures (Figure 1), we found both types of induced defocus (positive and negative) to significantly (*p* < 0.010) increase the size of perceived LD (Figures 1(a) and 1(b)) but not its irregularity (Figures 1(c) and 1(d)). Despite the nonsignificant differences in irregularity parameters of LD (*p* > 0.050, Wilcoxon paired samples test), we observed that positive defocus increased LD more than negative defocus.

### 3.2. Impact of HOA and Topographic Quality Parameters on Light Disturbance

**Figure 2:**
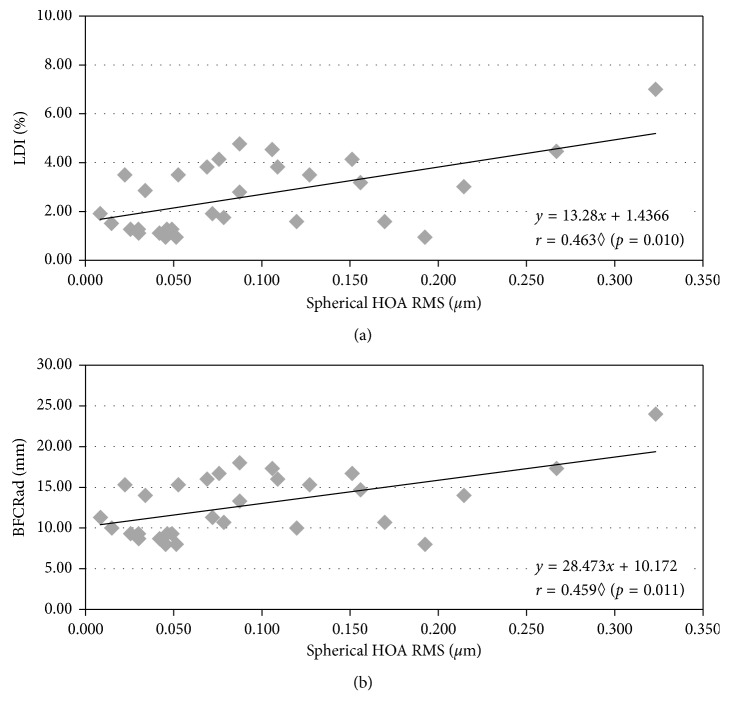
Correlations of spherical-like HOAs with LDI and BFCRad ((a) and (b), respectively) in baseline conditions. Removing the outlier does not change the strength of the correlation. Spearman correlation.

**Figure 3:**
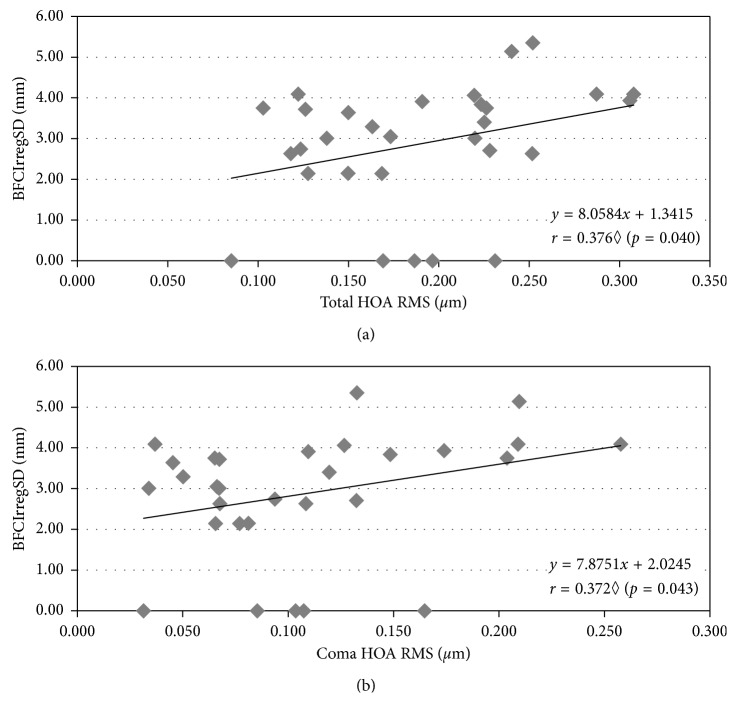
Correlations of total and coma-like HOAs with BFCIrregSD ((a) and (b), respectively) in cycloplegic conditions. Spearman correlation.

**Figure 4:**
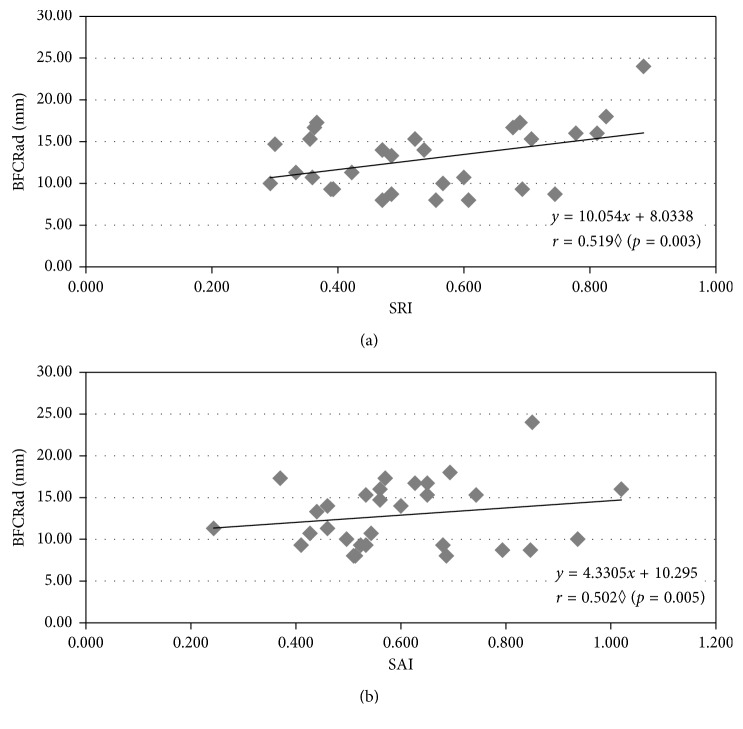
Correlations of the BFCRad with SRI (a) and SAI (b). Spearman correlation.

Figures 2–4 represent the more representative and statistically significant correlations of disturbance parameters with ocular aberrations and corneal regularity and symmetry parameters. The relationship between HOAs and LD parameters was evaluated to know how HOA can influence the perception of dysphotopsias. As seen in Figure 2, without cycloplegia, we found a moderate positive significant correlation between the total spherical-like HOAs and both size parameters of LD. By contrast, with cycloplegia, we found BFCIrregSD to be significantly correlated in a moderate positive way with total and coma-like HOAs (Figure 3).

Topographic parameters may have an influence on the image quality. We evaluated how topographic quality parameters, such as SAI, SRI, and Q-value, may influence the perception of photic phenomena in dim light conditions. A significant positive and moderate correlation between the BFCRad and both corneal surface indexes (SAI and SRI) was found (see Figure 4), but no significant correlation between any LD parameter and the Q-value (*r* ≤ 0.204, *p* ≥ 0.279, Spearman correlation).

## 4. Discussion

Previous studies [8, 16, 26, 28] reported that LD increases with positive defocus and described the changes in the retinal image quality when different levels of positive and negative defocus are induced. However, no studies were found to compare the psychophysical measure of LD under different levels of positive and negative defocus.

In the present study, we found monocular LD parameters to have higher values than binocular ones. These findings suggest that in binocular conditions, there is a neural capacity to attenuate the perceived LD. This improvement is consistent with several other studies showing a better visual performance in binocular conditions attributed to binocular summation when both eyes have similar visual acuities [39–41]. Plainis et al. [40] suggested that the attenuation of the defocus effect in binocular vision may be due to the activation of a major number of neurons close to the threshold detection. This has also been recently reported in multifocal intraocular and contact lens wearers [42, 43] with the same instrument to evaluate dysphotopsias. However, Jimenez et al. [39] showed that this summation effect might be impaired in subjects who underwent LASIK when the interocular differences in the vision quality are higher due to anisometropia and asymmetry in postsurgical corneal asphericity.

Comparing LD measures with and without cycloplegia, we observed that under paralyzed accommodation, the perception of LD was superior to natural conditions although the average pupil size under natural viewing was not significantly different from the 5 mm artificial aperture created under cycloplegic conditions. The same effect was observed in other study reporting the effect of different amounts of induced positive and negative spherical-like HOAs on LD in healthy subjects with a normal accommodative response and under cycloplegic conditions [25]. This may result from uncorrected latent hyperopic refractive errors revealed by the loss of tonic accommodation caused by the tropicamide effect. Moreover, these results suggest not only that active accommodation is capable of attenuating LD and minimizing the degradation of optical quality but also that defocus caused by low uncorrected refractive errors influences the perceived disturbances. In this study, positive and negative induced defocus increased the size of LD in a similar way, suggesting that uncorrected hyperopic and myopic refractive errors of the same level (1.00D in this study) should induce a LD of similar size. Although not significant, our results showed that negative induced defocus seems to shrink the size of LD compared to positive defocus. A potential explanation may be the incomplete accommodation paralyzation achieved with tropicamide that eventually allows subjects to slightly accommodate (about 0.5D), therefore partially compensating part of the negative induced defocus. Even without accommodating, when negative defocus is induced, positive spherical-like HOAs can be partially compensated, consequently reducing its effect. Macedo-de-Araújo et al. [25] previously found that inducing negative spherical-like HOA decreased the LD under the tropicamide effect, compared to those obtained with positive induced spherical-like HOAs and baseline. This suggests that the induction of negative spherical-like HOAs partially offsets the positive spherical-like HOAs intrinsic to each subject under these conditions.

Spherical defocus equally distorts the light source in all directions, which may be one of the causes for the insignificant changes in LD irregularity parameters with the induced defocus in this study. Therefore, the irregularity of LD did not change significantly in the present study. If those studies were performed with other types of induced defocus beyond spherical one, we would probably find differences in LD irregularity. In a previous study [26], we found astigmatic defocus to produce higher LD irregularity than the spherical one, with the corresponding meridional asymmetry.

The influence of the HOA magnitude on the visual quality is a well-known fact. Some studies explored the relationship between HOA and the perception of photic phenomena. In subjects who underwent LASIK surgery evaluated with Starlights v1.0, Villa et al. [13] found a significantly reasonable correlation between the halo disturbance index and corneal total, coma-like and spherical-like HOA RMS, as well as an increase in LD with the corneal HOA magnitude. We found significant positive correlations between the LD size and spherical-like HOAs, as well as between BFCIrregSD and total and coma-like HOAs, which agrees with the results of Villa et al. The present outcomes further suggest that spherical-like HOA is one of the main contributors to increase the LD size, while total and coma-like HOAs are related to the irregularity of LD.

Another objective of this study was to evaluate how topographic quality parameters SAI, SRI, and Q-value may influence LD. We found SAI and SRI to be positively correlated with BFCRad, an LD size parameter. These findings are in agreement with those found by Kojima et al. [44] for the glare score in subjects who underwent one month of orthokeratology treatment. We also found a significant correlation between SAI and BFCIrreg, which was not expected for normal (nontreated) corneas and might reflect LDA sensitivity to detect minor differences in LD related to topographical parameters within the normal range. On these grounds, it would be relevant to explore these associations in irregular eyes. By contrast, the Q-value did not show to be related to any LD parameter, suggesting that internal spherical aberrations might be more relevant for LD than the corneal spherical aberration itself (intrinsically related to the Q-value).

In summary, this study showed that the perception of LD might be influenced by the induction of both positive and negative defocus, binocularity, HOA, SAI, and SRI. Under cycloplegic conditions, the LD size increased similarly with both types of spherical defocus although lower uncorrected hyperopic refractive errors should be more carefully evaluated for these circumstances. The present results might be relevant to better understand the photic phenomena observed in the context of multifocal contact lens fitting and mainly in the context of refractive surgery, where small uncorrected refractive errors are a frequent outcome.

## Figures and Tables

**Table 1:** Demographic, refractive, and topographic characteristics of the sample (mean ± standard deviation).

Parameter	Description
*n*	30
Age (years)	23.28 ± 3.61
Gender	21 females (70%)
	9 males (30%)
M (D)	−0.56 ± 0.92
J0 (D)	0.07 ± 0.17
J45 (D)	−0.02 ± 0.13
Pupil size at mesopic conditions (mm)	5.64 ± 0.65
*Q*_mean_	0.28 ± 0.11
SIM *K*_mean_ (mm)	43.90 ± 1.44
IS index (D)	−0.21 ± 0.47
SRI	0.48 ± 0.15
SAI	0.60 ± 0.17

## Data Availability

The data used to support the findings of this study are available from the corresponding author upon request.
